# Thyroid Cancer in a Psoriatic Patient Treated With Secukinumab: A Case Report and Literature Review

**DOI:** 10.7759/cureus.93590

**Published:** 2025-09-30

**Authors:** Areej Alyousef, Hussein Attia, Mutlaq Alsulaili, Shehab Aldhafiri, Hamdy Sakr

**Affiliations:** 1 Dermatology, Al-Jahra Hospital, Kuwait, KWT; 2 Radiation Oncology, Kuwait Cancer Control Center, Kuwait, KWT

**Keywords:** autoimmune, biologics, cancer, complication of treatment, interleukin-17a, malignancy, medullary thyroid carcinoma, plaque, psoriasis, thyroid cancer

## Abstract

Biologics are a key treatment option for managing chronic inflammatory and autoimmune diseases, especially moderate to severe psoriasis. While studies have not definitively proven a causal link between biologics and cancer, concerns remain regarding their potential association with an increased risk of certain malignancies. This report describes an observed temporal association in a patient with psoriasis who was treated with the interleukin-17A inhibitor secukinumab for four years and subsequently developed metastatic medullary thyroid cancer.

## Introduction

Psoriasis is a chronic inflammatory autoimmune skin condition that can significantly impact a patient’s quality of life. The cutaneous manifestations of psoriasis are characterized by well-demarcated, erythematous, thick, scaly papules and plaques that may occur on the elbows, knees, or scalp, or may be widespread across the skin. These distinctive features arise from disrupted differentiation and proliferation of keratinocytes, resulting from the interaction between altered immune cells and keratinocytes in genetically susceptible individuals [1].

The estimated global prevalence of psoriasis is around 2-3% [2]. Approximately 20-30% of affected patients are expected to develop psoriatic arthritis during the course of their disease [3,4].

Common tools used to measure psoriasis severity include the Psoriasis Area and Severity Index (PASI), Body Surface Area (BSA), Dermatology Life Quality Index (DLQI), and Psoriasis Severity Scale (PSS). These tools aid in evaluating treatment efficacy and monitoring disease progression.

Biologics play a crucial role in the management of psoriasis, particularly for moderate to severe cases. They target specific molecular steps in the inflammatory cascade of immune-mediated pathogenesis, often resulting in significant symptom relief and improved quality of life. The main types of biologics available for psoriasis treatment include tumor necrosis factor (TNF) inhibitors, interleukin-17A inhibitors, and interleukin-23 inhibitors. These biologics have demonstrated significant efficacy in managing moderate to severe psoriasis and are selected based on individual patient needs [5].

Although secukinumab, an interleukin-17A inhibitor, has an overall favorable long-term safety profile, the reported malignancy risk of about 0.85% indicates that the risk is low but not absent [6]. In contrast, TNF inhibitors have been more strongly linked to an increased risk of certain cancers, particularly non-Hodgkin lymphoma [7].

Medullary thyroid carcinoma (MTC) is a rare and distinct form of thyroid cancer characterized by the production of calcitonin, a hormone secreted by the thyroid C-cells. It can present as sporadic, hereditary, or as part of syndromes such as Multiple Endocrine Neoplasia (MEN) type 2. MTC has no well-established link to psoriasis or interleukin inhibitors.

In this report, we present a case of MTC in a patient with psoriasis treated with secukinumab (Cosentyx), a monoclonal antibody targeting interleukin-17A.

## Case presentation

**Figure 1:**
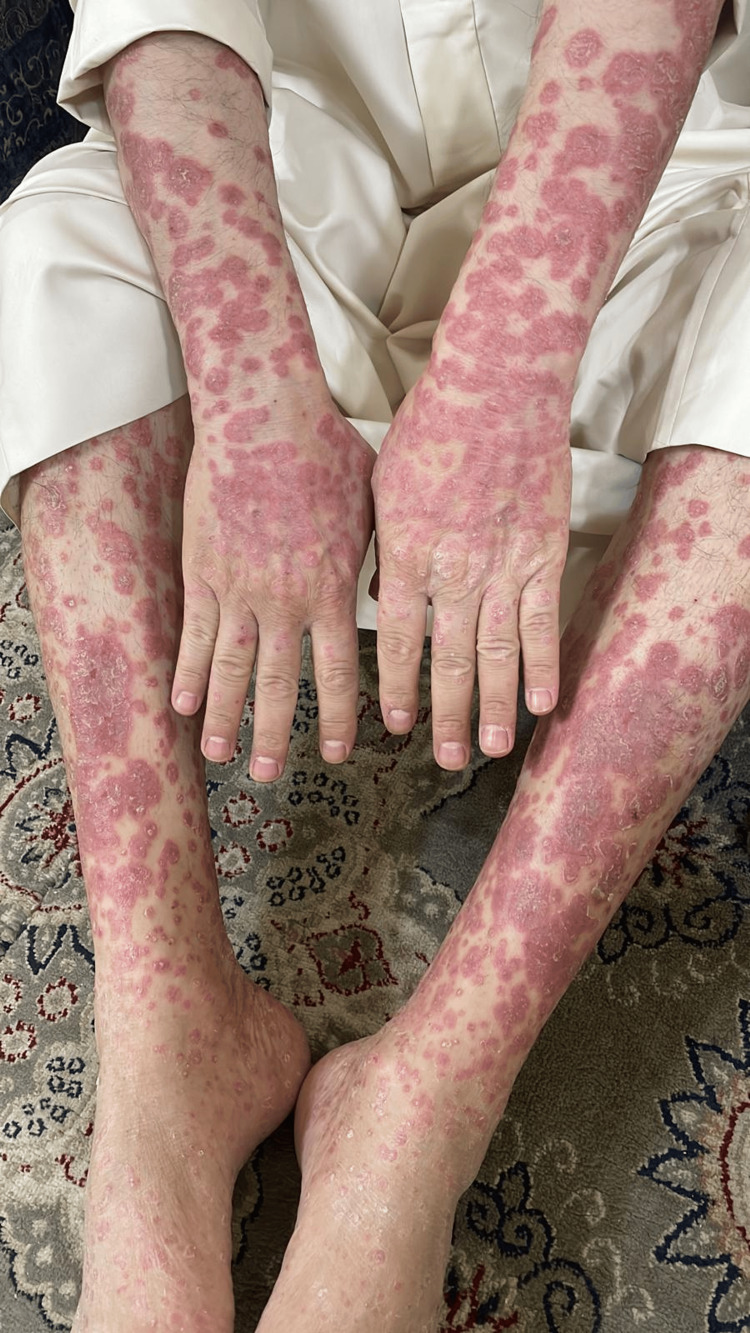
Multiple well-defined, rounded, closely related, and confluent erythematous scaly psoriatic plaques on the upper and lower extremities (before starting biologic therapy).

A 50-year-old gentleman, a heavy smoker with a 30-year history of severe plaque psoriasis (PASI score 20), presented with multiple psoriatic plaques involving the upper and lower limbs, trunk, scalp, and genitalia (Figure 1). The patient was started on the biologic therapy secukinumab (Cosentyx), an interleukin-17A inhibitor, in 2019, which resulted in significant improvement in his psoriatic lesions and a 95% reduction in his PASI score. However, he was diagnosed with medullary thyroid cancer (MTC) in February 2023 and had to discontinue the drug due to concerns about a potential causal relationship.

**Figure 2:**
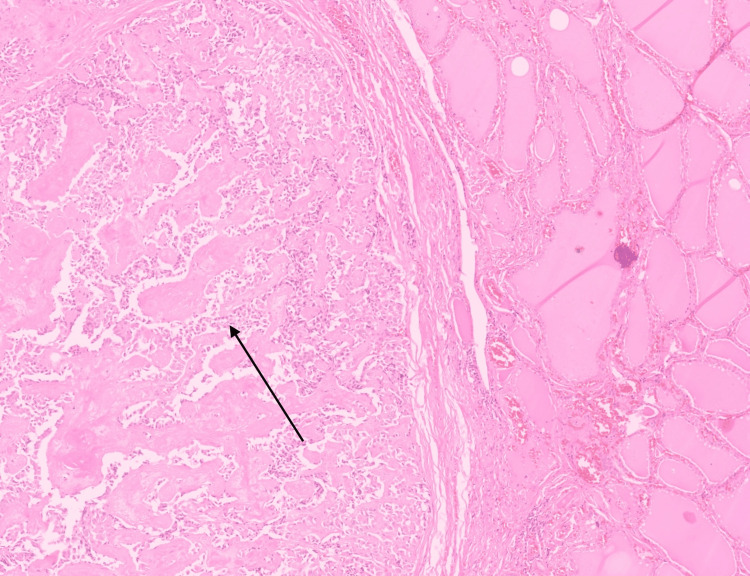
Thyroid tissue with adjacent areas of infiltration by medullary carcinoma (arrow shows area of infiltration). H&E stain, magnification ×10.

**Figure 3:**
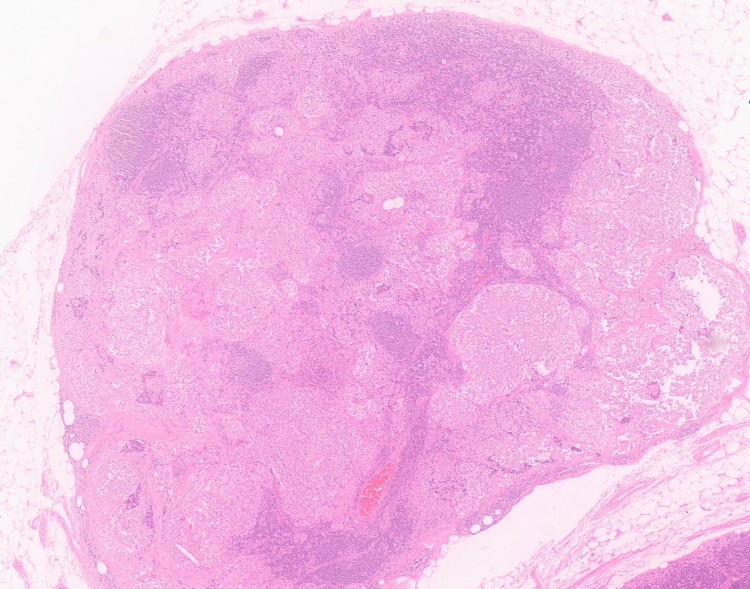
Lymph node with metastasis. H&E stain, magnification ×10.

**Figure 4:**
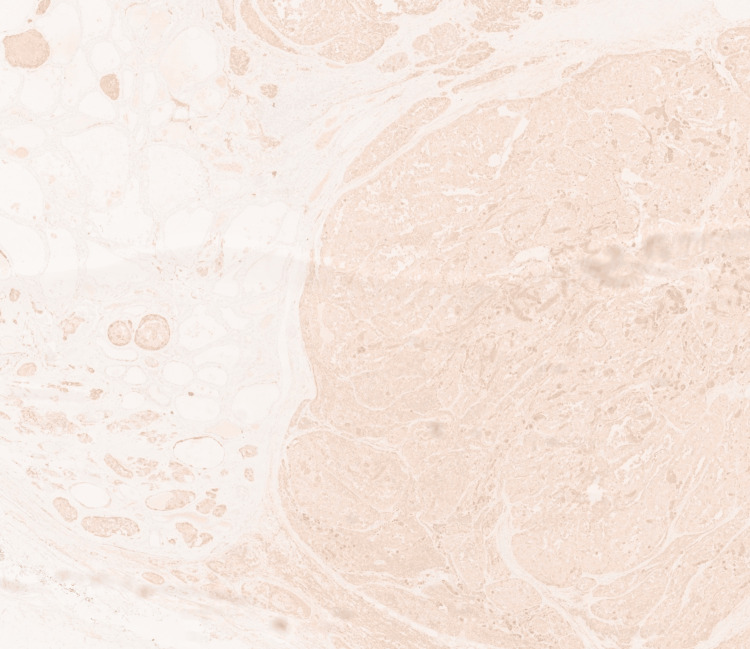
Positive calcitonin immunohistochemistry.

**Figure 5:**
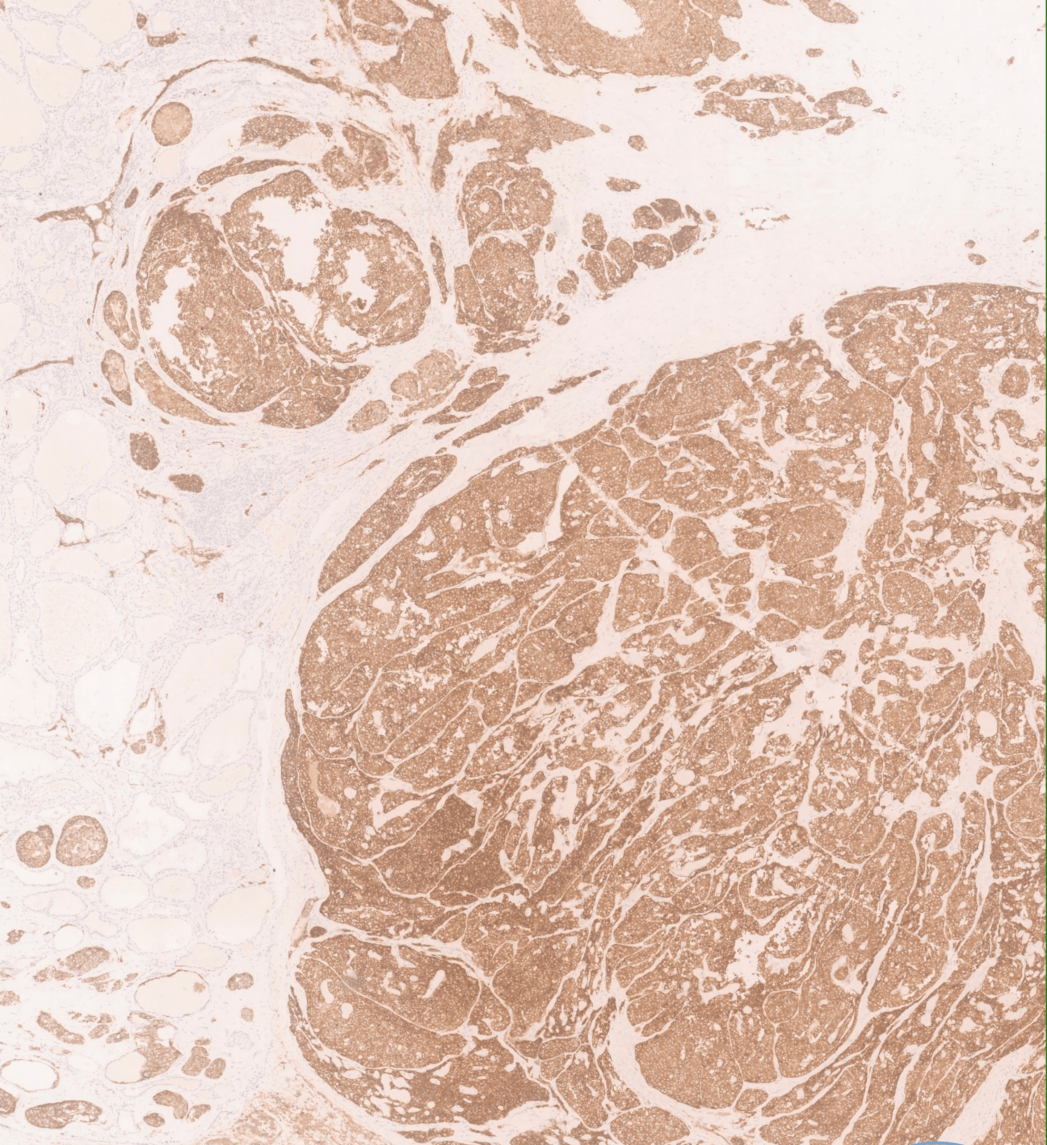
Positive synaptophysin immunohistochemistry.

The patient underwent total thyroidectomy with bilateral neck dissection in March 2023. Histopathologic examination revealed a unifocal lesion in the right thyroid lobe measuring 2 × 1.7 × 1.5 cm and lymph node metastasis with extra-nodal extension (Figures 2-3). Immunohistochemical evaluation showed tumor cells staining positive for CEA, synaptophysin, chromogranin, and calcitonin, while negative for thyroglobulin (Figures 4-5).

Final pathologic diagnosis: Sporadic RET-positive MTC, pT1bN1bM0.

This represented a sporadic, non-familial MTC, with a RET mutation identified exclusively in the tumor specimen (somatic). Genetic counseling and germline testing confirmed the absence of a hereditary RET mutation, and there was no reported family history of cancer.

His preoperative serum calcitonin level was 705 pg/mL; five weeks postoperatively the level dropped to 200 pg/mL. Postoperative radiologic assessment revealed no evidence of gross metastatic disease, and he was kept under close monitoring.

During follow-up, he experienced a severe relapse of his psoriatic lesions, which caused considerable distress. After discussion with the oncologist, the patient was subsequently started on the biologic guselkumab (Tremfya), an interleukin-23 inhibitor, in June 2023, achieving a 90% improvement in his PASI score.

**Figure 6:**
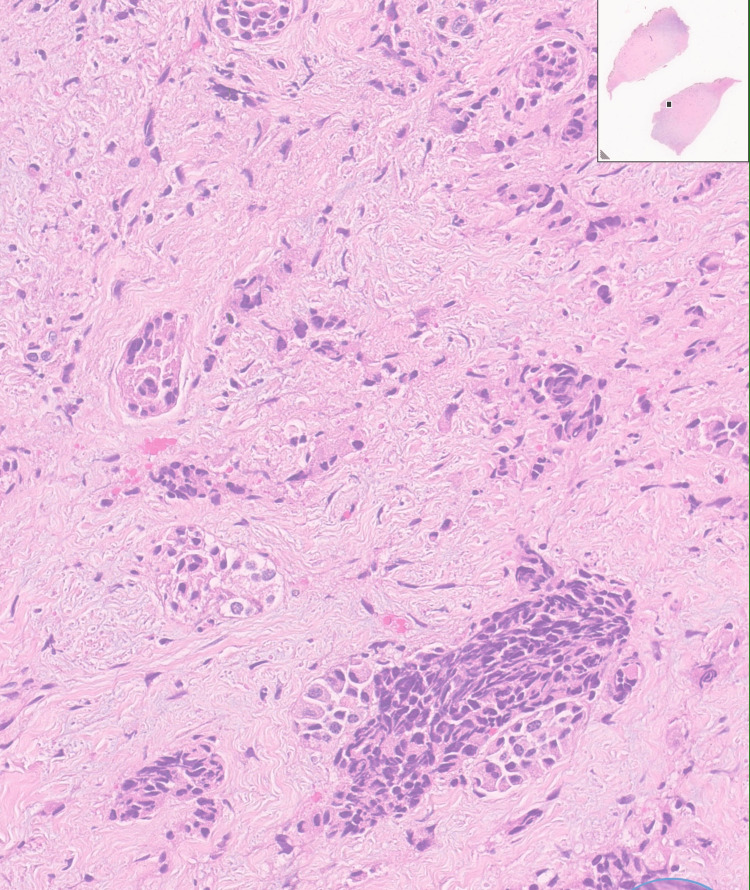
Hepatic parenchyma infiltrated by malignant cells. H&E stain, magnification ×40.

**Figure 7:**
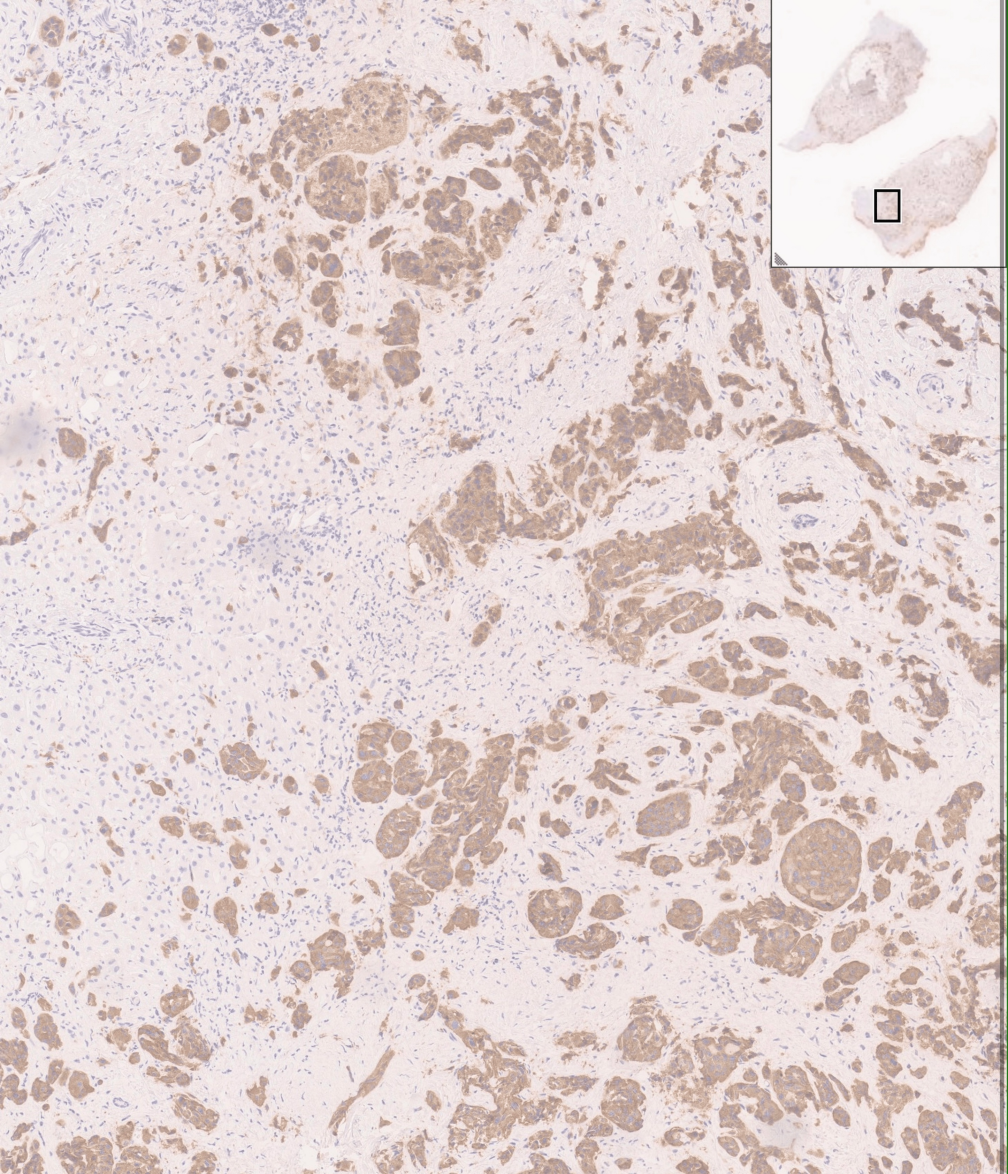
Liver metastasis positive for synaptophysin.

Incidentally, during a laparoscopic cholecystectomy in October 2023, the surgeon discovered a liver nodule, which was biopsied and identified as metastatic MTC (Figures 6-7).

A CT scan of the abdomen and pelvis revealed multiple hyperdense lesions in both lobes of the liver, suggestive of metastases, but no intrahepatic biliary radical dilation. Multiple enlarged retroperitoneal lymph nodes were present, with the largest located in the retro-pancreatic region, measuring approximately 2 × 3.3 cm. These findings were associated with a rising serum calcitonin level, reaching 700 pg/mL. Being asymptomatic at this stage, the patient was presented with the option to start treatment with an anti-RET kinase inhibitor, selpercatinib, or to undergo close follow-up. After a thorough discussion of the potential benefits and side effects of the medication, he chose regular follow-up.

A re-evaluation CT scan in May 2024 revealed a stationary course of the hepatic focal lesions and interval progression in both size and number of retro-pancreatic lymph nodes, along with newly identified mediastinal and bilateral hilar lymph nodes. Therefore, the oncology team decided to start the patient on selpercatinib 160 mg BID, which was eventually reduced to 80 mg BID due to severe toxicity in the form of rising serum creatinine, generalized joint pain, fatigue, and hypercalcemia, which developed four weeks after initiation of therapy.

Given the patient’s development of metastatic lesions from the primary MTC and the initiation of selpercatinib, both we and his oncologist decided to stop guselkumab (Tremfya) to avoid any potential risk of cancer reactivation or drug interaction. We then opted for narrowband UVB phototherapy to manage his psoriasis.

## Discussion

Biologic therapies have significantly transformed the management of chronic immune-mediated inflammatory conditions such as psoriasis, rheumatoid arthritis, and inflammatory bowel disease, owing to their high efficacy, sustained response, and favorable safety profile compared to conventional therapies. However, the relationship between biologic therapy and cancer risk is complex and continues to evolve, and it is increasingly recognized that not all biologic agents carry the same level of risk.

Currently, there are more than 10 biologic drugs approved by the FDA for moderate to severe plaque psoriasis. The first two drugs were approved in 2003: alefacept in January and efalizumab in October. These approvals marked major advancements in the treatment options available for this condition. However, both drugs were later withdrawn due to safety concerns, including the risk of progressive multifocal leukoencephalopathy (PML) [8].

Assessing the cancer risk linked to biologic therapies in psoriasis is challenging, as the already elevated cancer incidence in psoriatic patients makes it difficult to determine whether these treatments further increase that risk [9-11]. Several studies have explored this issue; however, the findings remain inconclusive, and more research is needed.

A case study by Shim DH et al. (2022) described a 58-year-old patient with psoriasis who was diagnosed with lung cancer after 62 months of treatment with secukinumab (Cosentyx). Consequently, the patient discontinued biologic therapy and switched to topical agents [12]. This case highlights the potential risks associated with long-term biologic therapy, but it remains unclear whether secukinumab directly contributed to the development of cancer.

Similarly, in 2018, Morizane S et al. reported a case of psoriasis in which the patient developed breast cancer after receiving systemic treatments including etretinate, cyclosporine, methotrexate, adalimumab, and ustekinumab. The case illustrated a potential association between biologic therapy and cancer development, although the authors noted that further research is needed to establish a causal relationship [13].

A recent study by Jung JM et al. (2023), using a nationwide population-based approach, found that TNF-α inhibitors in psoriatic patients were associated with a significantly higher risk of overall cancer and lymphoma [7]. This finding highlights the potential risks of certain biologics, particularly TNF-α inhibitors, in relation to cancer development.

While concerns about malignancy have been raised with biologic use, interleukin inhibitors are not associated with the same level of cancer risk as TNF-α inhibitors [7]. However, it is important to note that the available evidence remains limited, especially regarding rare cancers such as medullary thyroid carcinoma.

## Conclusions

The role of interleukin inhibitors and other biologic therapies in cancer development remains unresolved, with evidence particularly limited for rare malignancies such as medullary thyroid carcinoma. Ongoing vigilance and long-term studies are essential to better define the safety profiles of biologic agents, as no causal relationship can be established from a single case.
